# Cdk5 Phosphorylates Dopamine D2 Receptor and Attenuates Downstream Signaling

**DOI:** 10.1371/journal.pone.0084482

**Published:** 2013-12-31

**Authors:** Jaehoon Jeong, Young-Un Park, Dae-Kyum Kim, Saebom Lee, Yongdo Kwak, Seol-Ae Lee, Haeryun Lee, Yoo-Hun Suh, Yong Song Gho, Daehee Hwang, Sang Ki Park

**Affiliations:** 1 Department of Life Sciences, Pohang University of Science and Technology, Pohang, Republic of Korea; 2 School of Interdisciplinary Bioscience and Bioengineering, Pohang University of Science and Technology, Pohang, Republic of Korea; 3 Division of Integrative Bioscience and Biotechnology, Pohang University of Science and Technology, Pohang, Republic of Korea; 4 Korea Brain Research Institute, Daegu, Republic of Korea; University of North Dakota, United States of America

## Abstract

The dopamine D2 receptor (DRD2) is a key receptor that mediates dopamine-associated brain functions such as mood, reward, and emotion. Cyclin-dependent kinase 5 (Cdk5) is a proline-directed serine/threonine kinase whose function has been implicated in the brain reward circuit. In this study, we revealed that the serine 321 residue (S321) in the third intracellular loop of DRD2 (D2i3) is a novel regulatory site of Cdk5. Cdk5-dependent phosphorylation of S321 in the D2i3 was observed in *in vitro* and cell culture systems. We further observed that the phosphorylation of S321 impaired the agonist-stimulated surface expression of DRD2 and decreased G protein coupling to DRD2. Moreover, the downstream cAMP pathway was affected in the heterologous system and in primary neuronal cultures from p35 knockout embryos likely due to the reduced inhibitory activity of DRD2. These results indicate that Cdk5-mediated phosphorylation of S321 inhibits DRD2 function, providing a novel regulatory mechanism for dopamine signaling.

## Introduction

Dopamine signaling is involved in various brain functions including motor coordination, mood control and reward mechanisms [1]. A major component of dopamine signaling in vertebrates is exerted by striatal medium spiny neurons (MSNs) which selectively express a subset of dopamine receptors and receive dopaminergic input mainly from the ventral tegmental area (VTA) and substantia nigra (SN) [2]. Dopamine receptors are G protein-coupled receptors (GPCR) with seven transmembrane domains and consist of two subtypes, D1-like and D2-like receptors, that mediate reciprocal actions in dopamine signaling [1]. For example, dopamine D1-like receptors (D1, D5) activate adenylyl cyclase through G_αs_ and increase the intracellular level of cAMP, but dopamine D2-like receptors (D2, D3, D4) inhibit adenylyl cyclase through G_αi_ and decrease the intracellular level of cAMP [1], [3].

Among dopamine receptors, the D2 receptor (DRD2) is implicated in the pathophysiology of multiple major psychiatric disorders including schizophrenia and drug addiction [4], such that many antipsychotic drugs at least partially target DRD2. It is also known that DRD2 activity correlates well with the behavioral consequences of drugs of abuse in animal models [5]. Antidepressants and mood stabilizer efficacy have also been linked to alterations in the cell surface expression of DRD2 or downstream intracellular signaling mediated by PKA, ERK and GSK3 [1], [4], [6]. Despite these critical roles for DRD2 in the brain, the detailed regulatory mechanisms that confer heterogeneity and complexity to DRD2 properties are not completely understood.

Converging lines of evidence indicate that multiple posttranslational modifications are involved in the fine-tuning of DRD2 activity. Extensive glycosylation of DRD2 was revealed in early photo-affinity labeling studies [7], and disulfide bond formation within DRD2 was also identified as an important modification for ligand binding [8]. Furthermore, phosphorylation sites of DRD2 were initially identified by *in vitro* assay with radioisotopes, providing routes for various regulatory pathways mediated by various kinases [9]. Indeed, protein kinase C (PKC) regulates DRD2-mediated mobilization of intracellular calcium and modulates the interaction of DRD2 with cytoskeletal proteins [10]. Phosphorylation by GPCR kinase 2 (GRK2) regulates agonist-induced resensitization patterns of DRD2 [11].

Cyclin-dependent kinase 5 (Cdk5) is a proline-directed serine/threonine kinase that has preferential activity due to brain-specific expression of its essential activators, p35 and p39 [12]. Cdk5 is involved in various neuronal processes including neuronal migration and axon guidance, and Cdk5 and p35 null mice show defects in cortical layering [13]. Recently, it was shown that phosphorylation of WAVE1 and ephexin by Cdk5 regulates dendritic spine morphogenesis [14]. Furthermore, Cdk5 also regulates surface expression levels of the NMDA receptor, NR2B, and NR2A-mediated NMDA currents [15], [16]. It is noteworthy that multiple pieces of evidence suggest an intimate relationship between Cdk5 and the dopamine system. Cdk5 phosphorylates tyrosine hydroxylase (TH), regulating its stability, and thus maintaining dopaminergic homeostasis [17]. In postsynaptic neurons, when the T75 residue of dopamine and cyclic-AMP regulated phosphoprotein-32kD (DARPP-32) is phosphorylated by Cdk5, it can inhibit PKA activity and thus antagonize dopamine DRD1-mediated PKA signaling [18]. Interestingly, when cocaine, an indirect agonist of dopamine receptors, is administrated chronically in rats, mRNA and protein levels of Cdk5 increase in medium spiny neurons [19]. Collectively, Cdk5 appears to be involved in drug-induced synaptic adaptations. In this study, we show a functional interaction of DRD2 and Cdk5 that further extends the role of Cdk5 in dopamine signaling.

## Materials and Methods

### Antibodies

Anti-rabbit serums were raised against peptides containing phospho-serine 321 (pS321) of the third intracellular loop of DRD2 (D2i3). Phospho-peptide, CNPDpSPAKPEK (PEPTRON), was used to make a peptide-conjugated column for affinity purification (20401, PIERCE). Anti-pS321 antibody was enriched by an affinity purification system following the manufacturer’s instruction. Purified phospho-antibody was stored in PBS with 0.1% sodium azide and 0.1% gelatin. Anti-mouse anti-Cdk5 antibody (sc-249) and anti-rabbit anti-p35 antibody (sc-820) were used for the Western blotting and immunocytochemistry of Cdk5/p35. Anti-mouse anti-GFP antibody (sc-9996) was used for the immunoprecipitation and Western blotting of DRD2-GFP. Anti-rabbit anti-FLAG antibody (sc-807), anti-rabbit anti-HA antibody (sc-805), anti-mouse anti-GST antibody (sc-138), and anti-mouse anti-GAPDH antibody (sc-32293) were purchased from Santa Cruz Biotechnologies.

### Animals

The p35 knockout mouse was a kind gift from Dr. Katsuhiko Mikoshiba at RIKEN Brain Science Institute in Japan and used for primary neuron culture. Primer sets for genotyping were 5′- GGTCTCCTCTTCTGTCAAGAAG, 5′-GCTCTGCTAGACACATACTGTAC and 5′- TCCATCT GCACGAGACTAGT as previously described [20]. ICR mice and Sprague Dawley rats were used for brain lysate preparation. All animal procedures were approved by the Pohang University of Science and Technology Institutional Animal Care and Use Committee.

### Plasmid Constructs

Human DRD2 long isoform in an EGFP-N1 plasmid vector and the third intracellular loop of DRD2 (212–373 amino acid residues including the 29 additional amino acid residues unique to DRD2 long isoform) in a pFLAG-CMV-2 plasmid vector were used. Human Cdk5 was inserted in a pCMV-HA plasmid vector and human p35 was inserted in a pcDNA 3.1 plasmid vector. Human Cdk5 was inserted under a cytomegalovirus (CMV) promoter along with human p35 in a pcDNA 3.1 vector to make a dual expression construct (Cdk5/p35) for immunocytochemistry, receptor internalization assay, [^35^S]-GTP_γ_S binding assay, radioligand binding assay and cAMP enzyme immunoassay.

### *In Vitro* Kinase Assay

IP-linked *in vitro* kinase assay was performed as following. One whole mouse brain was lysed in 3 mL erythrocytes lysis buffer (ELB) (50 mM Tris (pH 8.0), 250 mM NaCl, 5 mM EDTA, 0.1% NP-40) by 20 strokes of a Dounce homogenizer to get homogenized brain lysates. The lysates were incubated on ice for 30 min, sonicated, and centrifuged at 16,000 × *g* for 10 min. The supernatants were immunoprecipitated with anti-rabbit anti-p35 antibody to obtain an active Cdk5/p35 complex. Cdk5/p35 complex and purified GST fusion protein was mixed with adenosine 5′-triposphate, [γ-^32^P] (NEG-502H, PerkinElmer) and incubated in kinase buffer (30 mM HEPES (pH 7.2), 10 mM MgCl_2_, 0.2 mM DTT) for 1 h at room temperature [18], [21]. Purified Cdk5/p25 complex (14–516, Millipore) was also used for *in vitro* kinase assay as described above. The 2× sample loading buffer was added to the reaction mixture and boiled at 100°C. The samples were then subjected to SDS-PAGE and the dried gel was assessed by autoradiography.

### Liquid Chromatography (LC)-Mass Spectrometry (MS)/MS Analysis

The recombinant GST-D2i3 protein was analyzed by LC-MS/MS following IP-linked *in vitro* kinase assay. We performed peptide identification of LC-MS/MS data using X!!Tandem (version Dec-01-2008). Each RAW data file was first converted to mzXML using the trans-proteomic pipeline (TPP; version 4.3). MS/MS scans in the converted mzXMLs were then subjected to search against the UniProt mouse protein sequence database (release 2010_07) including GST-D2i3 sequence using X!!Tandem. The tolerance was set to 3 Da for precursor ions and 2 Da for fragment ions. Enzyme specificity for trypsin was used. Variable modification options were used for the carbamidomethylation of cysteine (57.021 Da), the oxidation of methionine (15.995 Da), the hydrolysis of asparagine (0.987 Da) and the phosphorylation of serine (79.966 Da).

### Immunoprecipitation

Immunoprecipitation was performed on cell lysates in ELB lysis buffer. Anti-GFP antibody was added to the lysates and incubated for 3 h at 4°C. Immunocomplexes were purified using protein-A agarose. The precipitates were incubated with SDS sample loading buffer for 30 min at 37°C, and subjected to SDS-PAGE and Western blots.

### GST Pull-down Assay

10 µg of purified GST and GST–D2i3 were incubated with rat brain lysate for 1.5 h at 4°C. 30 µL of glutathione (GSH)-conjugated Sepharose 4B beads (17-0756-01, GE Healthcare) equilibrated with lysis buffer was added and incubated for additional 1 h. Beads were collected by centrifugation at 2,000×*g* and rinsed with lysis buffer 4 times [22], [23]. Precipitates were analyzed by Western blotting using anti-Cdk5 and anti-p35 antibodies.

### Immunocytochemistry

Transfected HEK 293 cells and striatal neurons cultured on coverslips were washed once with phosphate buffered saline (PBS) and fixed by immersion in cold 4% paraformaldehyde/PBS for 30 min. Primary antibody was diluted in the blocking solution (2% horse serum and 1% Triton X-100 in PBS). Alexafluor-647-conjugated anti-mouse antibody (A20990, Invitrogen) and Alexafluor-568-conjugated anti-rabbit antibody (A11011, Invitrogen) were used as secondary antibodies. Hoechst were used for nucleus staining. Images were obtained by confocal microscopy (Olympus, FluoView-1000).

### Receptor Internalization Assay

24 h after transfection, cells were treated with 1 µM quinpirole (Q102, Sigma) for 30 min and 90 min at 37°C. Cells were re-suspended in 2 mL cold PBS and 200 µL aliquots were used for each reaction. Drug treatments were carried out at room temperature for 3 h at the following concentrations; 3 nM [^3^H]-spiperone (NET-565, PerkinElmer), 3 µM sulpiride (895, TOCRIS), 10 µM haloperidol (H1512, Sigma). Hydrophobic [^3^H]-spiperone was used to label total expressed receptors and hydrophilic sulpiride was used to replace membranous receptor-bound [^3^H]-spiperone signals. Membrane-associated receptor signals were calculated by subtracting intracellular receptor values from the total expressed receptor values. Cells were filtered on a GF/B (Millipore) filter and washed 3 times with washing buffer (50 mM Tris-HCl (pH 7.4), 100 mM NaCl). Filters were dried out and residual radioactivity was measured using a liquid scintillation counter [24].

### Cell Membrane Preparation

Confluent cells in 100 mm culture-dishes after transfection were washed with ice-cold PBS and harvested in 1 mL HME buffer (25 mM HEPES (pH 7.5), 2 mM MgCl_2_, 1 mM EDTA). Homogenized lysates were centrifuged with 500×g for 15 min and the supernatants were subsequently centrifuged with 36,000×g for 30 min. Pellets re-suspended in HME buffer were used for assays.

### [^35^S]-GTP_γ_S Binding Assay

Cell membrane fractions were pre-incubated with 1 µM quinpirole (Q102, Sigma) in the assay buffer (25 mM HEPES (pH 7.5), 1.5 mM MgCl_2_, 100 mM NaCl, 1 mM EDTA and 0.01 mM GDP) for 10 min. [^35^S]-GTP_γ_S (NET-030H, PerkinElmer) was added to the final concentration of 3 nM in 30 µL and further incubated for 90 min. 170 µL of ice-cold buffer (10 mM Tris-HCl (pH 8.0), 100 mM NaCl, 10 mM MgCl_2_, and 0.1 mM GTP) was added to stop the reaction. Membranes were filtered on a GF/B filter (Millipore) and washed 3 times with washing buffer (50 mM Tris-HCl (pH 7.4), 100 mM NaCl). Filters were dried and radioactivity was measured using the scintillation counter [25], [26].

### Radioligand Binding Assay

Prepared cell membranes were incubated with 0.01 nM [^3^H]-spiperone (NET-565, PerkinElmer) and increasing concentrations of quinpirole (Q102, Sigma) for 30 min in the assay buffer (25 mM HEPES (pH 7.5), 1.5 mM MgCl_2_, 100 mM NaCl, 1 mM EDTA). Membranes were filtered on a GF/B filter (Millipore) and washed 3 times with washing buffer (50 mM Tris-HCl (pH 7.4), 100 mM NaCl). The reaction was terminated by rapid filtration through GF/C filters. Residual radioactivity was measured using a liquid scintillation counter [27]–[29].

### cAMP Enzyme Immunoassay

Transfected HEK 293 cells were pretreated with 10 µM rolipram (R6520, Sigma) for 1 h, and then treated with 0.1 µM forskolin (F6886, Sigma) and increasing concentrations of quinpirole (Q102, Sigma) for 30 min. Primary cultured striatal neurons were treated with 10 µM rolipram for 1 h, and then 1 µM dopamine for 1 h [22]. Cell lysates were prepared with 0.1 M HCl and cAMP levels were detected by cAMP enzyme immunoassay kit (Sapphire Bioscience) following the manufacturer’s instruction.

### Primary Cultured Striatal Neuron

Striatal area was isolated from the mouse embryonic brain (E15). Dissected tissue was dissociated in minimal essential media (MEM) (11095, Invitrogen) containing 0.25% trypsin (T4549-100, Sigma) and 0.1% DNase I for 6 min at 37°C. Cells were re-suspened in the plating media (MEM with 0.01 M HEPES (pH 7.4) and 10% (vol/vol) horse serum (16050-122, GIBCO)). Neurons were cultured for 7 days *in vitro* (DIV 7) in MEM with B27 supplement (17504-044, Invitrogen) before being applied to cAMP enzyme immunoassays.

## Results

### Cdk5 Phosphorylates Serine 321 in the Third Intracellular Loop of DRD2 *in vitro*

**Figure 1:**
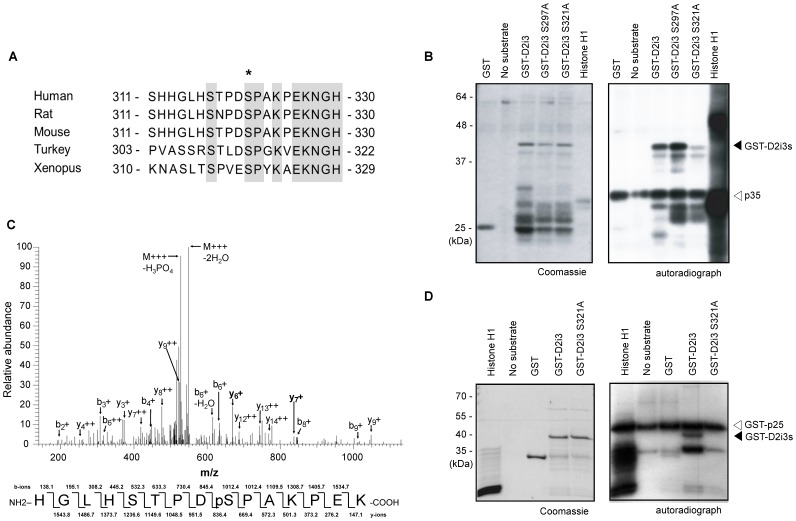
Cdk5 phosphorylates serine 321 in the third intracellular loop of DRD2 *in vitro.* (A) Amino acid sequence alignment showing conserved regions of the DRD2 from various species (shaded). The potential Cdk5 phosphorylation site is indicated by an asterisk. (B) IP-linked *in vitro* kinase assay with recombinant GST-D2i3 and GST-D2i3 mutant proteins. Cdk5/p35 complex enriched from mouse brain extract by anti-p35 immunoprecipitation was used for kinase reactions. An autoradiograph of phosphorylated proteins is shown along with Coomassie brilliant blue staining of the same gel. Arrowhead indicates radioactive signal corresponding to GST-D2i3s and open arrowhead indicates radioactive signals from p35. (C) MS/MS spectrum of the phosphorylated peptide fragment of D2i3. The theoretical fragmentation patterns are shown below the spectrum. Among all the fragment ions, the detected y- and b-ions are denoted in the spectrum. The y_6_ and y_7_ ions strongly indicate the phosphorylation of serine 321. (D) *In vitro* kinase assay with purified Cdk5/GST-p25 complex using GST-D2i3 and GST-D2i3 mutant proteins. Phosphorylated proteins were shown in an autoradiograph, along with Coomassie brilliant blue staining. Arrowhead indicates radioactive signal corresponding GST-D2i3 and open arrowhead indicates radioactive signals from GST-p25.

To identify novel Cdk5 substrates, we performed a systematic search using (S/T)PX(K/H/R) as the Cdk5 recognition consensus sequences [30] and identified DRD2 as a candidate substrate. The consensus sequence, including serine 321, is located in the third intracellular loop of DRD2 (D2i3) where various regulatory mechanisms have been implicated [3], [10], [11]. The sequence is evolutionarily conserved in DRD2 in vertebrates, implying a functional importance of the residue (Fig. 1A).

To assess the capacity of Cdk5 to phosphorylate D2i3, we performed IP-linked *in vitro* kinase assays using an active Cdk5/p35 complex enriched from mouse brain lysate by p35 immunoprecipitation with purified recombinant GST-D2i3 (amino acid residues 212–373) proteins as the substrates. We observed phosphorylation signals in the purified GST-D2i3 and GST-D2i3 S297A proteins, but the signal was significantly diminished using GST-D2i3 S321A (Fig. 1B). To further verify phosphorylation of serine 321 in the GST-D2i3, we performed LC-MS/MS analysis of the samples from IP-linked *in vitro* kinase assays using LTQ XL mass spectrometry. Consistently, phospho-peptides corresponding to the mass of phospho-serine 321 peptides were recovered (Fig. 1C). Considering that the data-dependent acquisition during LC-MS/MS analysis tends to detect abundant proteins in the sample [31], this data suggests that the serine 321 residue is the dominant phosphorylation site of Cdk5 in the D2i3 region. To prove direct phosphorylation of serine 321 in the GST-D2i3 by Cdk5, *in vitro* kinase assay using purified Cdk5/GST-p25 complex with purified recombinant GST-D2i3 proteins was performed. We identified a significant phosphorylation signal in the GST-D2i3 that was absent in the GST-D2i3 S321A (Fig. 1D). Taken together, these results indicate that the D2i3 S321 residue is a preferential target for Cdk5-mediated phosphorylation.

### Cdk5 Phosphorylates Serine 321 in the Third Intracellular Loop of DRD2 in Cells

**Figure 2:**
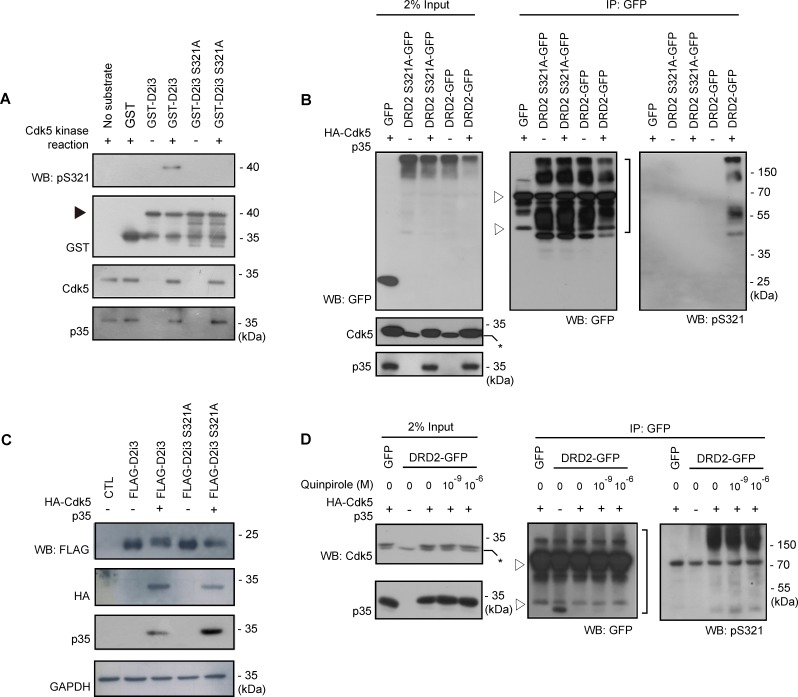
Cdk5 phosphorylates serine 321 in the third intracellular loop of DRD2 in cells. Cdk5-mediated phosphorylation of serine 321 was analyzed using anti-pS321 antibody. (A) Samples from IP-linked *in vitro* kinase assay using GST-D2i3 proteins were analyzed by Western blotting (WB) with indicated antibodies. Arrowheads indicate GST-D2i3s. (B) DRD2-GFP and DRD2 S321A-GFP was expressed with or without HA-Cdk5 and p35 in HEK 293 cells. Anti-GFP immunoprecipitates were analyzed by Western blotting using anti-GFP and anti-pS321 antibodies. The bracket indicates DRD2 signals and open arrowhead indicates nonspecific signals from the anti-GFP immunoprecipitates. ‘% input’ is % volume of total lysate for an IP reaction. Weak endogenous Cdk5 signals were indicated by asterisks. (C) Gel mobility shift assay. HEK 293 cells transfected as indicated were analyzed by Western blotting. (D) Transfected HEK 293 cells were treated with quinpirole and anti-GFP immunoprecipitates were analyzed by Western blotting with anti-GFP and anti-pS321 antibodies. Open arrowhead indicates nonspecific signals from anti-GFP immunoprecipitates.

To identify the phosphorylation of serine 321, we raised antibody specific for phospho-serine 321 (pS321). Samples from the IP-linked *in vitro* kinase assay were analyzed by Western blotting using anti-pS321 antibody. Blots showed a distinct band in the kinase reaction that was dependent on GST-D2i3 (Fig. 2A). To verify the potential phosphorylation of serine 321 in DRD2 by Cdk5 in cells, anti-GFP immunoprecipitates from HEK 293 cells expressing DRD2-GFP and DRD2 S321A-GFP with or without HA-Cdk5 and p35 were analyzed by Western blotting using anti-GFP and anti-pS321 antibodies. Characteristic smeared band signals by anti-GFP antibody that are known to be due to excessive glycosylation of DRD2 are observed only in the presence of DRD2-GFP, and anti-pS321 antibody detected similar DRD2 signals only with Cdk5/p35 co expression (Fig. 2B) [7]. To further verify the phosphorylation of serine 321 by Cdk5, D2i3 (FLAG-D2i3) and mutant form of D2i3 (FLAG-D2i3 S321A) were generated. FLAG-D2i3 and FLAG-D2i3 S321A expressed with or without HA-Cdk5 and p35 in HEK 293 cells were analyzed by an SDS-gel mobility shift assay. A significant Cdk5-dependent mobility shift was observed for FLAG-D2i3, but not for FLAG-D2i3 S321A (Fig. 2C). We also assessed the phosphorylation level of DRD2 at Ser321 upon agonist stimulation. HEK 293 cells expressing DRD2-GFP and Cdk5/p35 complex were stimulated by quinpirole, and anti-GFP immunoprecipitates from the cell lysates were analyzed by Western blotting using anti-GFP and anti-pS321 antibodies. We found that Cdk5-mediated phosphorylation of DRD2 at Ser321 was not affected by agonist stimulation, which appears different from GRK- and PKC-mediated phosphorylations (Fig. 2D) [32], [33]. Together, these results indicate that Cdk5 can phosphorylate the serine 321 residue of DRD2 in the cellular environment.

### Cdk5/p35 Complex and DRD2 are Physically Associated

**Figure 3:**
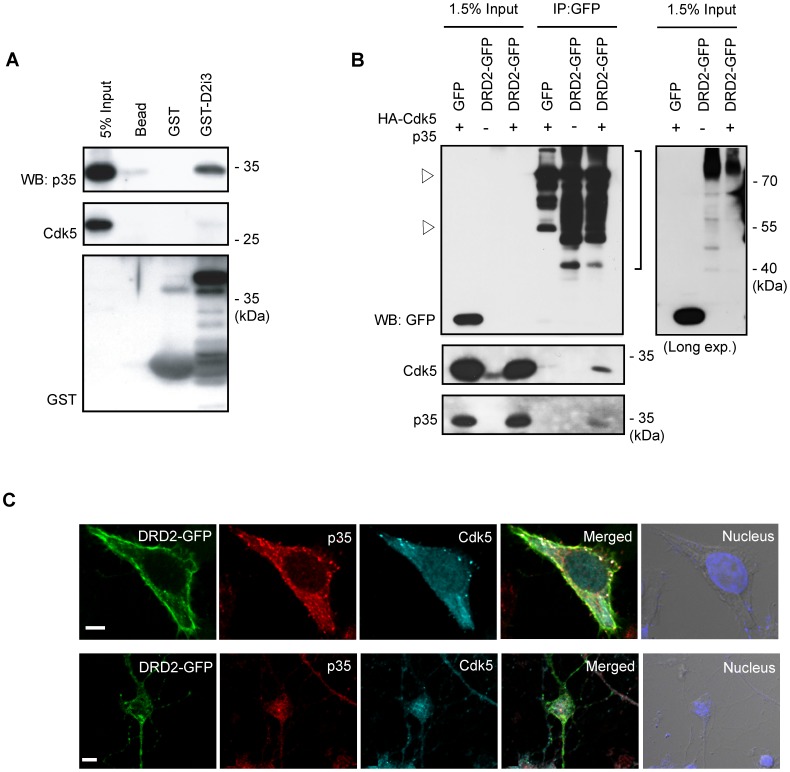
Cdk5/p35 can form a complex with DRD2. (A) GST pull-down assay using purified recombinant GST-D2i3 protein with rat brain extract. GST pull-down precipitates were subjected to Western blotting analyses. ‘Bead’ indicates the pull-down precipitate without GST proteins. (B) Immunoprecipitation of DRD2 and Cdk5/p35 complex. Anti-GFP IP from lysates from transfected cells were subjected to Western blotting analyses. The bracket indicates DRD2 signals and open arrowhead indicates nonspecific signals from the anti-GFP immunoprecipitates. An overexposed blot for inputs is also shown in the right. (C) Immunocytochemical analyses of DRD2 and Cdk5/p35. HEK 293 cells expressing DRD2-GFP and Cdk5/p35 were stained with anti-Cdk5 and anti-p35 antibodies (Upper panels). DRD2-GFP was expressed alone in the cultured striatal neurons and stained with anti-Cdk5 and anti-p35 antibodies (Lower panels). Hoechst were used for nucleus staining. The scale bar is 5 µm. All images were obtained using confocal microscopy (Olympus, FluoView-1000).

We investigated the potential physical interaction between the Cdk5/p35 complex and DRD2 because many Cdk5 substrates are known to be physically associated with Cdk5/p35 complex [23], [34], [35]. First, the GST pull-down experiment was performed. Purified recombinant GST-D2i3 protein was incubated with rat brain lysate and GST pull-down precipitates were analyzed for Western blotting. As shown in Fig. 3A, endogenous Cdk5 and p35 were identified in the pull-down precipitates, indicating a physical interaction between DRD2 and the Cdk5/p35 complex (Fig. 3A). Moreover, HA-Cdk5 and p35 were detected in the anti-GFP immunoprecipitates from HEK 293 cell lysates expressing DRD2-GFP and Cdk5/p35 (Fig. 3B). In addition, we performed immunocytochemical analyses and observed that DRD2-GFP, HA-Cdk5 and p35 show significant co-localization signals at the membranous area of HEK 293 cells (Fig. 3C, upper panels). We also investigated co-localization of DRD2 and Cdk5/p35 in the neuronal context. Consistently, DRD2-GFP also showed significant co-localization with endogenous Cdk5 and p35 in the cultured striatal neurons (DIV7), further supporting functional links between DRD2 and Cdk5/p35 (Fig. 3C, bottom panels). The results indicate that DRD2 and Cdk5/p35 can form a complex and thus, support the notion that DRD2 is a physiological substrate of Cdk5.

### Cdk5-mediated Phosphorylation of DRD2 Attenuates Receptor Activity

**Figure 4:**
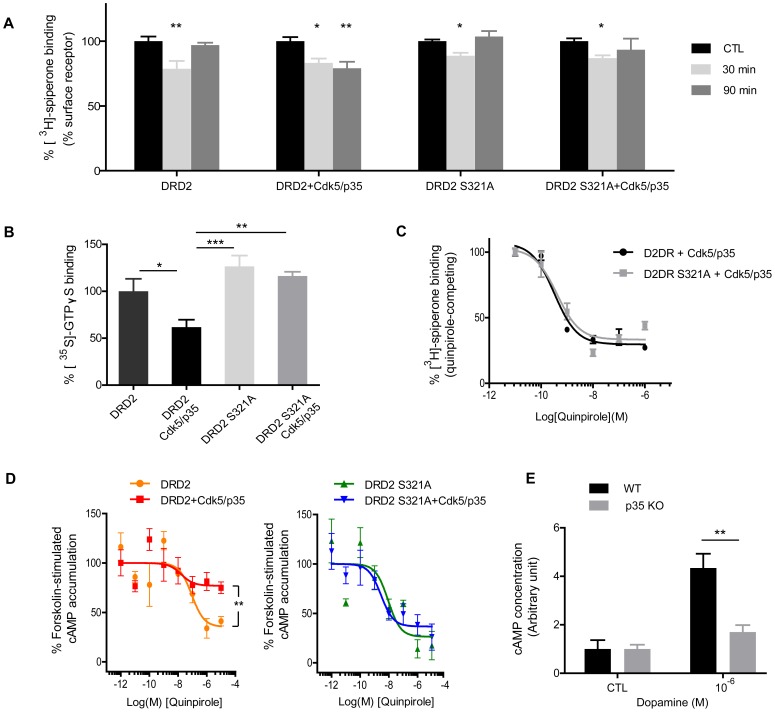
Cdk5-mediated phosphorylation attenuates DRD2 surface expression and downstream signaling. (A) DRD2 surface expression measured by [^3^H]-spiperone binding assay. Transfected HEK293 cells were stimulated with 1 µM quinpirole for the indicated time and harvested, followed by 3 nM [^3^H]-spiperone treatment for 3 h. Radioactivity was measured and surface signals were calculated. Error bars represent mean ± SE (n = 8; *p<0.05, **p<0.01; One-way ANOVA with Dunnett post hoc test: compare all columns vs. control column). (B) [^35^S]-GTP_γ_S binding assay. Cell membranes were prepared from the cells transfected as indicated. Membrane preparations were incubated with 1 µM quinpirole followed by 3 nM [^35^S]-GTP_γ_S for 90 min. Error bars represent mean ± SE (n = 8; *p<0.05, **p<0.01, ***p<0.001; One-way ANOVA with Bonferroni post hoc test: compare all pairs of columns). (C) Quinpirole-competing [^3^H]-spiperone binding assay. Membrane preparations from transfected cells were incubated with 0.01 nM [^3^H]-spiperone and increasing concentrations of quinpirole for 30 min. Non-linear regression was obtained by GraphPad. Error bars indicate mean ± SE (n = 3). (D) cAMP enzyme immunoassays in transfected HEK 293 cells. Transfected cells were pretreated with 10 µM rolipram for 1 h, and subsequently co-treated with 0.1 µM forskolin and increasing concentrations of quinpirole for 30 min. Non-linear regression was obtained by GraphPad. Error bars represent mean ± SE (n = 4; **p<0.01; two-tailed *t*-tests). (E) Cultured striatal neurons from wild type and p35 knockout embryos (DIV 7) were treated with 10 µM rolipram for 1 h followed by 1 µM dopamine for 1 h. Error bars represent mean ± SE (n = 4; **p<0.01; two-tailed *t*-tests).

It has been reported that phosphorylation modulates critical properties of GPCRs such as G protein coupling, receptor internalization, intracellular localization, and association with modulator proteins [9], [11], [24]. Agonist-induced receptor internalization is a critical regulatory process of signal transduction. We investigated Cdk5-mediated modulation of DRD2 internalization. HEK 293 cells expressing DRD2-GFP and DRD2 S321A-GFP with or without Cdk5/p35 were incubated with 1 µM quinpirole to induce agonist-stimulated DRD2 internalization (Fig. 4A). [^3^H]-spiperone signals of DRD2-GFP expressing cells were significantly reduced at 30 min quinpirole treatment and recovered at 90 min. Interestingly, [^3^H]-spiperone signals of DRD2-GFP and Cdk5/p35 expressing cells were also reduced at 30 min quinpirole treatment but not recovered at 90 min (Fig. 4A, second section). On the other hand, [^3^H]-spiperone signals of DRD2 S321A-GFP expressing cells were reduced at 30 min and recovered at 90 min, regardless of the co-expression with Cdk5/p35. Previous studies have shown that the internalized DRD2 recycles back to the plasma membrane upon prolonged agonist stimulation [11]. Thus it appears that Cdk5-mediated phosphorylation of DRD2 is involved in the resensitization processes following agonist-induced DRD2 internalization.

We further evaluated a potential change of agonist-stimulated G protein coupling to DRD2 associated with Cdk5-mediated phosphorylation using [^35^S]-GTP_γ_S binding assay [25], [26]. DRD2-GFP and DRD2 S321A-GFP with or without Cdk5/p35 were expressed in HEK 293 cells. Membranes were prepared and stimulated with 1 µM quinpirole and further allowed [^35^S]-GTP_γ_S incorporation. DRD2-GFP and Cdk5/p35 expressing cell membrane showed significantly impaired [^35^S]-GTP_γ_S binding compared to all the other cell membranes (Fig. 4B). These results indicate that Cdk5-mediated phosphorylation down-regulates agonist-stimulated G protein binding at DRD2.

Additionally, quinpirole-competing [^3^H]-spiperone binding assays were performed to investigate any potential change in agonist-affinity at DRD2 by Cdk5-mediated phosphorylation. Competitive binding of [^3^H]-spiperone upon treatment of increasing concentrations of quinpirole to the membrane preparation from transfected was measured. Competing binding of quinpirole and [^3^H]-spiperone at DRD2-GFP and DRD2 S321A-GFP made similar logK_i_ values (−9.789 for DRD2-GFP; −9.691 for DRD2 S321A-GFP), indicating that the affinity of ligand to DRD2 is not significantly affected by Cdk5-mediated phosphorylation at DRD2 (Fig. 4C).

### Cdk5-mediated Phosphorylation Down-regulates the DRD2-cAMP Signaling Pathway

Next, we investigated whether the modification of DRD2 by Cdk5 affects downstream signaling pathways. We monitored DRD2-mediated inhibition of forskolin-stimulated cAMP production by adenylyl cyclase in the cells expressing DRD2-GFP and DRD2 S321A-GFP using cAMP enzyme immunoassay. DRD2-expressing cells showed decreased cAMP levels in response to quinpirole in a dose-dependent manner. Remarkably, co-expression of Cdk5/p35 significantly reduced the maximal inhibition of cAMP formation (Fig. 4D, left panel). On the other hand, in the DRD2 S321A-GFP expressing cells, the cAMP formations were effectively inhibited in response to quinpirole treatment regardless of the expression of Cdk5/p35 (Fig. 4D, right panel). These results indicate that Cdk5-mediated phosphorylation of DRD2 attenuates the inhibitory activity of DRD2 on the downstream cAMP signaling pathway. To further confirm the phenomena in a more physiologically relevant setting, we made use of primary cultured neurons from knockout embryos deficient in p35, an essential Cdk5 activator. Primary cultured striatal neurons were treated with 1 µM dopamine and analyzed by cAMP enzyme immunoassay. Neurons from p35 knockout mice exhibited reduced cAMP levels compared to wild-type neurons when stimulated with dopamine (Fig. 4E). Taken together, we concluded that Cdk5-mediated phosphorylation of DRD2 results in a decrease in the inhibitory tone on the cAMP pathway exerted by DRD2.

## Discussion

We identified DRD2 as a novel substrate of Cdk5. The phosphorylation appears to down-regulates DRD2 surface expression by affecting the fate of DRD2 following receptor internalization thereby reducing DRD2 G_i_-coupling and downstream cAMP pathway. As the phosphorylation residue S321 exists both in DRD2 long and short isoforms, the mechanism proposed in this study may be a general mode of regulation in DRD2-mediated signaling.

DRD2 in medium spiny neurons has not only been regarded as a major dopamine receptor subtype but has also been recognized for its susceptibility to changes in availability in response to environmental stimuli. Agonist-induced desensitization and resensitization of DRD2 have been extensively studied [11], [24]. In particular, a number of studies have shown that the effects of chronic psychostimulant exposure, such as cocaine and amphetamine, which raise the extracellular level of dopamine in the striatal synapse, are accompanied by dynamic changes of DRD2 postsynaptically [36]. Indeed, chronic cocaine users are known to have reduced DRD2 levels in the striatal area, and DRD2 availability in the nucleus accumbens (NAcc) shows a negative correlation with the drug seeking and reinforcement behaviors in mice and primates [37]–[39]. These findings indicate that the functionality of DRD2 is highly susceptible to adaptive or compensatory regulation in response to various stimuli including chronic drug exposure. Our results show that the S321 residue in the third intracellular loop of DRD2 can be phosphorylated by Cdk5, which results in a decrease in inhibitory influence of DRD2 on the cAMP pathway. This interaction proposes a novel regulatory mechanism associated with Cdk5 in dopaminoceptive neurons that might be linked to the dynamic nature of DRD2 surface availability.

It should be noted that Cdk5 is known to be a key component in mediating adaptive changes of the neuronal environment. For instance, structural and functional alterations of dendritic spines in the neurons of the limbic circuit are one of the consequences of repeated psychostimulant exposure [40]. These changes are accompanied by various molecular changes including the induction of cAMP response element-binding protein (CREB) and ΔFosB, transcription factors that exhibit an enduring up-regulation in response to chronic cocaine administration [41], [42]. Importantly, Cdk5 is a target of ΔFosB [19], and many critical components involved in dendritic spine dynamics, such as PSD-95, p21-activated kinase (PAK), β-catenin, and spinophilin, were reported as Cdk5 substrates [43]–[46]. Consistently, genetic or pharmacological manipulations of Cdk5 activity elicit alterations of dendritic spine morphology and behavioral responses to cocaine, implying critical roles for Cdk5 in the molecular and morphological changes of mesolimbic dopamine circuits [47], [48]. Our results showing that DRD2 is a novel target of Cdk5 provides additional insight into the adaptive changes of the dopamine system in response to chronic drug exposures because of the subsequent ΔFosB-mediated up-regulation of Cdk5 may induce a tonic increase in the phosphorylation of DRD2. Moreover, DRD2 is known to affect numerous cellular processes, including regulation of cAMP and MAP kinase pathways and downstream transcriptional events [42], [49]. Thus, the findings in this study might not only depict a direct regulation of DRD2 by Cdk5 but also provide a novel insight into the adaptive responses of dopamine system to chronic drug exposure.

## References

[1] Missale C, Nash SR, Robinson SW, Jaber M, Caron MG (1998) Dopamine receptors: from structure to function. Physiol Rev 78: 189–225. 9457173 10.1152/physrev.1998.78.1.189

[2] Wise RA (2002) Brain reward circuitry: insights from unsensed incentives. Neuron 36: 229–240. 12383779 10.1016/s0896-6273(02)00965-0

[3] Neve KA, Seamans JK, Trantham-Davidson H (2004) Dopamine receptor signaling. J Recept Signal Transduct Res 24: 165–205. 15521361 10.1081/rrs-200029981

[4] Amar S, Shaltiel G, Mann L, Shamir A, Dean B, et al (2008) Possible involvement of post-dopamine D2 receptor signalling components in the pathophysiology of schizophrenia. Int J Neuropsychopharmacol 11: 197–205. 17681085 10.1017/S1461145707007948

[5] Caine SB, Negus SS, Mello NK, Patel S, Bristow L, et al (2002) Role of dopamine D2-like receptors in cocaine self-administration: studies with D2 receptor mutant mice and novel D2 receptor antagonists. J Neurosci 22: 2977–2988. 11923462 10.1523/JNEUROSCI.22-07-02977.2002 PMC6758322

[6] Lee S, Jeong J, Park YU, Kwak Y, Lee SA, et al (2012) Valproate alters dopamine signaling in association with induction of Par-4 protein expression. PLoS One 7: e45618. 23029138 10.1371/journal.pone.0045618 PMC3454414

[7] Fishburn CS, Elazar Z, Fuchs S (1995) Differential glycosylation and intracellular trafficking for the long and short isoforms of the D2 dopamine receptor. J Biol Chem 270: 29819–29824. 8530376 10.1074/jbc.270.50.29819

[8] Reader TA, Molina-Holgado E, Lima L, Boulianne S, Dewar KM (1992) Specific [3H]raclopride binding to neostriatal dopamine D2 receptors: role of disulfide and sulfhydryl groups. Neurochem Res 17: 749–759. 1379349 10.1007/BF00969008

[9] Ng GY, O’Dowd BF, Caron M, Dennis M, Brann MR, et al (1994) Phosphorylation and palmitoylation of the human D2L dopamine receptor in Sf9 cells. J Neurochem 63: 1589–1595. 7931316 10.1046/j.1471-4159.1994.63051589.x

[10] Li M, Bermak JC, Wang ZW, Zhou QY (2000) Modulation of dopamine D(2) receptor signaling by actin-binding protein (ABP-280). Mol Pharmacol 57: 446–452. 10692483 10.1124/mol.57.3.446

[11] Cho D, Zheng M, Min C, Ma L, Kurose H, et al (2010) Agonist-induced endocytosis and receptor phosphorylation mediate resensitization of dopamine D(2) receptors. Mol Endocrinol 24: 574–586. 20160122 10.1210/me.2009-0369 PMC2840813

[12] Tsai LH, Delalle I, Caviness VS Jr, Chae T, Harlow E (1994) p35 is a neural-specific regulatory subunit of cyclin-dependent kinase 5. Nature 371: 419–423. 8090221 10.1038/371419a0

[13] Dhavan R, Tsai LH (2001) A decade of CDK5. Nat Rev Mol Cell Biol 2: 749–759. 11584302 10.1038/35096019

[14] Fu AK, Ip NY (2007) Cyclin-dependent kinase 5 links extracellular cues to actin cytoskeleton during dendritic spine development. Cell Adh Migr 1: 110–112. 19270534 10.4161/cam.1.2.4617 PMC2633981

[15] Wang J, Liu S, Fu Y, Wang JH, Lu Y (2003) Cdk5 activation induces hippocampal CA1 cell death by directly phosphorylating NMDA receptors. Nat Neurosci 6: 1039–1047. 14502288 10.1038/nn1119

[16] Zhang S, Edelmann L, Liu J, Crandall JE, Morabito MA (2008) Cdk5 regulates the phosphorylation of tyrosine 1472 NR2B and the surface expression of NMDA receptors. J Neurosci 28: 415–424. 18184784 10.1523/JNEUROSCI.1900-07.2008 PMC6670547

[17] Moy LY, Tsai LH (2004) Cyclin-dependent kinase 5 phosphorylates serine 31 of tyrosine hydroxylase and regulates its stability. J Biol Chem 279: 54487–54493. 15471880 10.1074/jbc.M406636200

[18] Bibb JA, Snyder GL, Nishi A, Yan Z, Meijer L, et al (1999) Phosphorylation of DARPP-32 by Cdk5 modulates dopamine signalling in neurons. Nature 402: 669–671. 10604473 10.1038/45251

[19] Bibb JA, Chen J, Taylor JR, Svenningsson P, Nishi A, et al (2001) Effects of chronic exposure to cocaine are regulated by the neuronal protein Cdk5. Nature 410: 376–380. 11268215 10.1038/35066591

[20] Ohshima T, Ogawa M, Veeranna, Hirasawa M, Longenecker G, et al (2001) Synergistic contributions of cyclin-dependant kinase 5/p35 and Reelin/Dab1 to the positioning of cortical neurons in the developing mouse brain. Proc Natl Acad Sci U S A 98: 2764–2769. 11226314 10.1073/pnas.051628498 PMC30213

[21] Morabito MA, Sheng M, Tsai LH (2004) Cyclin-dependent kinase 5 phosphorylates the N-terminal domain of the postsynaptic density protein PSD-95 in neurons. J Neurosci 24: 865–876. 14749431 10.1523/JNEUROSCI.4582-03.2004 PMC6729809

[22] Park SK, Nguyen MD, Fischer A, Luke MP, Affar el B, et al (2005) Par-4 links dopamine signaling and depression. Cell 122: 275–287. 16051151 10.1016/j.cell.2005.05.031

[23] Niethammer M, Smith DS, Ayala R, Peng J, Ko J, et al (2000) NUDEL is a novel Cdk5 substrate that associates with LIS1 and cytoplasmic dynein. Neuron 28: 697–711. 11163260 10.1016/s0896-6273(00)00147-1

[24] Kim KM, Valenzano KJ, Robinson SR, Yao WD, Barak LS, et al (2001) Differential regulation of the dopamine D2 and D3 receptors by G protein-coupled receptor kinases and beta-arrestins. J Biol Chem 276: 37409–37414. 11473130 10.1074/jbc.M106728200

[25] Waldhoer M, Bofill-Cardona E, Milligan G, Freissmuth M, Nanoff C (1998) Differential uncoupling of A1 adenosine and D2 dopamine receptors by suramin and didemethylated suramin (NF037). Mol Pharmacol 53: 808–818. 9584206

[26] Bofill-Cardona E, Kudlacek O, Yang Q, Ahorn H, Freissmuth M, et al (2000) Binding of calmodulin to the D2-dopamine receptor reduces receptor signaling by arresting the G protein activation switch. J Biol Chem 275: 32672–32680. 10926927 10.1074/jbc.M002780200

[27] List SJ, Seeman P (1981) Resolution of dopamine and serotonin receptor components of [3H]spiperone binding to rat brain regions. Proc Natl Acad Sci U S A 78: 2620–2624. 6941314 10.1073/pnas.78.4.2620 PMC319401

[28] Gardner B, Strange PG (1998) Agonist action at D2(long) dopamine receptors: ligand binding and functional assays. Br J Pharmacol 124: 978–984. 9692784 10.1038/sj.bjp.0701926 PMC1565475

[29] Seeman P, Tallerico T, Ko F (2003) Dopamine displaces [3H]domperidone from high-affinity sites of the dopamine D2 receptor, but not [3H]raclopride or [3H]spiperone in isotonic medium: Implications for human positron emission tomography. Synapse 49: 209–215. 12827639 10.1002/syn.10232

[30] Obenauer JC, Cantley LC, Yaffe MB (2003) Scansite 2.0: Proteome-wide prediction of cell signaling interactions using short sequence motifs. Nucleic Acids Res 31: 3635–3641. 12824383 10.1093/nar/gkg584 PMC168990

[31] Liu H, Sadygov RG, Yates JR 3rd (2004) A model for random sampling and estimation of relative protein abundance in shotgun proteomics. Anal Chem 76: 4193–4201. 15253663 10.1021/ac0498563

[32] Ito K, Haga T, Lameh J, Sadee W (1999) Sequestration of dopamine D2 receptors depends on coexpression of G-protein-coupled receptor kinases 2 or 5. Eur J Biochem 260: 112–119. 10091590 10.1046/j.1432-1327.1999.00125.x

[33] Namkung Y, Sibley DR (2004) Protein kinase C mediates phosphorylation, desensitization, and trafficking of the D2 dopamine receptor. J Biol Chem 279: 49533–49541. 15347675 10.1074/jbc.M408319200

[34] Wong AS, Lee RH, Cheung AY, Yeung PK, Chung SK, et al (2011) Cdk5-mediated phosphorylation of endophilin B1 is required for induced autophagy in models of Parkinson’s disease. Nat Cell Biol 13: 568–579. 21499257 10.1038/ncb2217

[35] Kesavapany S, Lau KF, McLoughlin DM, Brownlees J, Ackerley S, et al (2001) p35/cdk5 binds and phosphorylates beta-catenin and regulates beta-catenin/presenilin-1 interaction. Eur J Neurosci 13: 241–247. 11168528

[36] Kuhar MJ, Ritz MC, Boja JW (1991) The dopamine hypothesis of the reinforcing properties of cocaine. Trends Neurosci 14: 299–302. 1719677 10.1016/0166-2236(91)90141-g

[37] Volkow ND, Fowler JS, Wolf AP, Schlyer D, Shiue CY, et al (1990) Effects of chronic cocaine abuse on postsynaptic dopamine receptors. Am J Psychiatry 147: 719–724. 2343913 10.1176/ajp.147.6.719

[38] Dalley JW, Fryer TD, Brichard L, Robinson ES, Theobald DE, et al (2007) Nucleus accumbens D2/3 receptors predict trait impulsivity and cocaine reinforcement. Science 315: 1267–1270. 17332411 10.1126/science.1137073 PMC1892797

[39] Nader MA, Morgan D, Gage HD, Nader SH, Calhoun TL, et al (2006) PET imaging of dopamine D2 receptors during chronic cocaine self-administration in monkeys. Nat Neurosci 9: 1050–1056. 16829955 10.1038/nn1737

[40] Robinson TE, Kolb B (1997) Persistent structural modifications in nucleus accumbens and prefrontal cortex neurons produced by previous experience with amphetamine. J Neurosci 17: 8491–8497. 9334421 10.1523/JNEUROSCI.17-21-08491.1997 PMC6573726

[41] Robinson TE, Kolb B (1999) Alterations in the morphology of dendrites and dendritic spines in the nucleus accumbens and prefrontal cortex following repeated treatment with amphetamine or cocaine. Eur J Neurosci 11: 1598–1604. 10215912 10.1046/j.1460-9568.1999.00576.x

[42] McClung CA, Nestler EJ (2003) Regulation of gene expression and cocaine reward by CREB and DeltaFosB. Nat Neurosci 6: 1208–1215. 14566342 10.1038/nn1143

[43] Feng J, Yan Z, Ferreira A, Tomizawa K, Liauw JA, et al (2000) Spinophilin regulates the formation and function of dendritic spines. Proc Natl Acad Sci U S A 97: 9287–9292. 10922077 10.1073/pnas.97.16.9287 PMC16860

[44] Prange O, Murphy TH (2001) Modular transport of postsynaptic density-95 clusters and association with stable spine precursors during early development of cortical neurons. J Neurosci 21: 9325–9333. 11717366 10.1523/JNEUROSCI.21-23-09325.2001 PMC6763916

[45] Murase S, Mosser E, Schuman EM (2002) Depolarization drives beta-Catenin into neuronal spines promoting changes in synaptic structure and function. Neuron 35: 91–105. 12123611 10.1016/s0896-6273(02)00764-x

[46] Hayashi ML, Choi SY, Rao BS, Jung HY, Lee HK, et al (2004) Altered cortical synaptic morphology and impaired memory consolidation in forebrain- specific dominant-negative PAK transgenic mice. Neuron 42: 773–787. 15182717 10.1016/j.neuron.2004.05.003

[47] Benavides DR, Quinn JJ, Zhong P, Hawasli AH, DiLeone RJ, et al (2007) Cdk5 modulates cocaine reward, motivation, and striatal neuron excitability. J Neurosci 27: 12967–12976. 18032670 10.1523/JNEUROSCI.4061-07.2007 PMC6673301

[48] Meyer DA, Richer E, Benkovic SA, Hayashi K, Kansy JW, et al (2008) Striatal dysregulation of Cdk5 alters locomotor responses to cocaine, motor learning, and dendritic morphology. Proc Natl Acad Sci U S A 105: 18561–18566. 19017804 10.1073/pnas.0806078105 PMC2587606

[49] Impey S, Obrietan K, Storm DR (1999) Making new connections: role of ERK/MAP kinase signaling in neuronal plasticity. Neuron 23: 11–14. 10402188 10.1016/s0896-6273(00)80747-3

